# Structural Characterization of Disulfide-Linked p53-Derived Peptide Dimers

**DOI:** 10.21203/rs.3.rs-4644285/v1

**Published:** 2024-07-19

**Authors:** Magdalena C. DiGiorno, Nisansala Vithanage, Clara G. Victorio, Dale F. Kreitler, Victor K. Outlaw, Nicholas Sawyer

**Affiliations:** Fordham University; University of Missouri; Fordham University; National Synchrotron Light Source II; University of Missouri; Fordham University

**Keywords:** Dimerization, Disulfide, Structure, Folding

## Abstract

Disulfide bonds provide a convenient method for chemoselective alteration of peptide and protein structure and function. We previously reported that mild oxidation of a p53-derived bisthiol peptide (CTFANLWRLLAQNC) under dilute non-denaturing conditions led to unexpected disulfide-linked dimers as the exclusive product. The dimers were antiparallel, significantly α-helical, resistant to protease degradation, and easily reduced back to the original bisthiol peptide. Here we examine the intrinsic factors influencing peptide dimerization using a combination of amino acid substitution, circular dichroism (CD) spectroscopy, and X-ray crystallography. CD analysis of peptide variants suggests critical roles for Leu6 and Leu10 in the formation of stable disulfide-linked dimers. The 1.0 Å resolution crystal structure of the peptide dimer supports these data, revealing a leucine-rich LxxLL dimer interface with canonical knobs-into-holes packing. Two levels of higher-order oligomerization are also observed in the crystal: an antiparallel “dimer of dimers” mediated by Phe3 and Trp7 residues in the asymmetric unit and a tetramer of dimers mediated by Trp7 and Leu10. In CD spectra of Trp-containing peptide variants, minima at 227 nm provide evidence for the dimer of dimers in dilute aqueous solution. Importantly, and in contrast to the original dimer model, the canonical leucine-rich core and robust dimerization of most peptide variants suggests a tunable molecular architecture to target various proteins and evaluate how folding and oligomerization impact various properties, such as cell permeability.

## Introduction

Disulfide bonds play a critical role in the structures and functions of both natural and designed proteins and peptides. Disulfide bonds covalently link distal regions within a wide range of peptides, including oxytocin (Gimpl & Fahrenholz, 2001; Hammer et al., 2023; Muttenthaler et al., 2010), cyclotides (de Veer et al., 2019; Northfield et al., 2014), disulfide-rich peptides (Singh et al., 2010), insulin (Karas et al., 2021), and proteins (Dombkowski et al., 2014; Mitchinson & Wells, 1989; Pace et al., 1988; Zavodszky et al., 2001), including antibodies (Goto & Hamaguchi, 1979; McAuley et al., 2008; Thies et al., 2002). Three-dimensional folding imposed by disulfide bonds is critical for the structure and function of these molecules, many of which have significant clinical value.

Thus, synthetic strategies for regioselective disulfide bond formation have been the subject of intensive study, especially in peptides and proteins containing multiple cysteine (Cys) residues (Albericio et al., 1991; Andreu et al., 1994; Postma & Albericio, 2014). Oxidative folding conditions can be sufficient in some cases but tend to require long reaction times to proceed to thermodynamic equilibrium and/or avoid kinetic traps (Shi et al., 2022; Welker et al., 2001). Orthogonal protecting groups are frequently used to allow formation of one disulfide bond at a time, including “one-pot” strategies (Laps et al., 2021; Postma et al., 2012; Spears et al., 2021). Alternatively, substitution of cysteine with analogs like penicillamine (Pen) favors selective Cys-Pen disulfide bond formation and can allow regioselective disulfide bond formation (Yao et al., 2022; Zheng et al., 2015).

Recently, Victorio and Sawyer reported the surprising, spontaneous formation of disulfide-linked dimers from a p53-derived bisthiol peptide called CV1 (CTFANLWRLLAQNC) using a mild aqueous buffer and dimethyl sulfoxide (DMSO) as oxidant (Victorio & Sawyer, 2023). This peptide is derived from SAH-p53–8, a stapled variant of the N-terminal segment of the p53 protein that interacts with the proteins Mdm2 and MdmX (Baek et al., 2012; Bernal et al., 2010). The interactions between p53 and Mdm2 and/or MdmX are major targets for drug discovery, especially in cancers with Mdm2 and/or MdmX upregulation (Chandramohan et al., 2024; Chang et al., 2013; Momand et al., 1998; Silvestri et al., 2023; Vassilev et al., 2004; Zhu et al., 2022). Beyond the challenge of creating stable peptides that bind tightly and specifically to Mdm2 and/or MdmX, the delivery of p53-derived peptides into target cells remains a significant challenge. Intracellular delivery is impacted by many factors, including amino acid composition, three-dimensional structure, and oligomerization state (Baek et al., 2012; Bernal et al., 2007; Chang et al., 2013; Dantas de Araujo et al., 2018; Henchey et al., 2010; Yoo et al., 2020).

In initial studies, dimers of the CV1 peptide were determined to be exclusively antiparallel with no detectable formation of competing parallel dimers or intramolecular disulfide species. Dimerization was dependent on α-helical folding of CV1, yielding highly α-helical dimers. To account for an unusual minimum observed at 227 nm by circular dichroism (CD) spectroscopy, a model of the dimer was proposed in which tryptophan residues interact at the dimer interface, which is uncommon but not unprecedented in coiled-coil dimers (Rhys et al., 2021). This model accounted for the fact that interactions between aromatic residues frequently give rise to CD maxima/minima in the 225–230 nm wavelength range (Andersen et al., 2006; Cochran et al., 2001; Nazzaro et al., 2023; Sawyer & Arora, 2018).

Here we describe structural characterization of CV1 dimers using a combination of alanine substitution, CD spectroscopy, and X-ray crystallography. Antiparallel dimers were readily produced from all CV1 variants except the L6A variant, which lacked α-helical character as a bisthiol monomer. Dimers displayed a range of α-helical character, and most dimers retained their maximal α-helical structure up to 70°C. The 1.0 Å crystal structure of the CV1 dimer revealed a knobs-into-holes packing interface between leucine residues at the dimer interface. The formation of a “dimer of dimers” arrangement in the asymmetric unit and a higher order “tetramer of dimers” is facilitated by Phe and Trp residues. These data revise the previous model of CV1 oligomerization and suggest a designable molecular architecture for potential targeting of a wide variety of proteins and studying how peptide folding and oligomerization influence interactions with proteins, nucleic acids, and membranes.

## Materials and Methods

### General Information.

Commercially purchased solvents and reagents were used without further purification. Nα-Fmoc-protected amino acids and peptide synthesis reagents were purchased from Advanced ChemTech, ChemImpex International, Oakwood Chemical, and Gyros Protein Technologies. Peptides were synthesized manually or using a Gyros Protein Technologies PurePep^™^ Chorus synthesizer. Peptides were purified on preparative C_18_ columns using reverse-phase high-performance liquid chromatography (RP-HPLC) on a Shimadzu Nexera HPLC system using gradients of water and acetonitrile (ACN) containing 0.1% trifluoroacetic acid (TFA). Peptide purity was evaluated by analytical HPLC using a Luna 5 μm C18(2) column (150 × 4.6 mm) on an Agilent 1100 HPLC system with a flow rate of 0.3 mL/min (gradient:5–95% solvent B over 30 minutes, solvent A = 0.1% TFA, B = 95% ACN, 5% water, 0.1% TFA, see Figure S1, Table S1). High-resolution mass spectrometry data was collected on a Bruker autoflex maX MALDI-TOF/TOF mass spectrometer using α-cyano-4-hydroxycinnamic acid as the matrix. Proteinase K (P8107S) was purchased from New England Biolabs. Mass spectrometry grade trypsin was purchased from Fisher (PI90057).

### Peptide Synthesis.

Peptides were synthesized following standard Fmoc solid-phase approaches on high-loading Rink MBHA resin (0.62 mmol/g resin). Peptides were globally deprotected and cleaved from resin by incubation with Reagent R (90% TFA, 5% thioanisole, 3% 1,2-ethanedithiol, and 2% anisole) for 2 h. After filtration, rotary evaporation of TFA and precipitation with ice-cold diethyl ether yielded crude peptides. A list of bisthiol peptides used for these studies is provided in Table 1.

### Thiol Oxidation Reactions.

Thiol oxidation reactions were performed by sequentially adding aqueous NH_4_HCO_3_ (pH 6.0), DMSO, and peptide to produce a solution with final concentrations of 0.88 mM peptide, 20% DMSO, 5.5 mM NH_4_HCO_3_, pH 6.0 (Tam et al., 1991). Reactions were performed at room temperature for 24 h. After each reaction, dimers were purified by RP-HPLC using a gradient of 25–70% ACN over 30 min.

### Circular Dichroism.

Circular dichroism spectra were acquired using a Jasco J-1500 CD spectrometer at a concentration of 30 μM in 10 mM sodium phosphate, pH 7.5 in a 0.1 cm pathlength cell. Unless specified, all spectra were collected at 25°C. For temperature-dependent circular dichroism experiments, CD spectra were acquired at 5°C intervals starting at a minimum temperature of 5°C and ending at a maximum temperature of 95°C. After acquisition of each spectrum, the sample temperature was increased at a rate of 1°C/min and held at the new target temperature for a minimum of 60 s before acquisition of the next spectrum.

### Trypsin Digestion.

Trypsin digestion samples were prepared by combining 10 μL of 300 μM peptide in water, 40 μL of 125 μM phosphate buffer (pH 7.2), and 10 μL of 1.2 μM trypsin in phosphate buffer at 37°C. Mass spectrometry data were collected after 15 h.

### X-ray Crystallography.

A crystallization stock solution was prepared containing the dimeric CV1 peptide in water at a concentration of 36 mg/mL. Crystals were grown by hanging drop vapor diffusion using a crystallization condition optimized from Molecular Dimensions Morpheus crystallization screen (30 mM sodium nitrate, 30 mM dibasic sodium phosphate, 30 mM ammonium sulfate, 20% (v/v) glycerol, 10% (w/v) PEG4000, 100 mM imidazole/MES monohydrate buffer, pH 6.5. A 2 μL drop comprising a 1:1 mixture of crystallization stock solution and the crystallization condition was placed on a glass cover slide and inverted to seal a well containing 150 μL of the crystallization condition. Crystal morphology is shown in Figure S2. Crystals were looped and vitrified in liquid nitrogen. Diffraction data were collected at the AMX beamline (17-ID-1) at the National Synchrotron Light Source II (NSLS-II) at Brookhaven National Laboratory (Upton, NY). Data were indexed, integrated, and scaled using the program *XDS* (Kabsch, 2010) and merged using the program *Aimless* (Evans & Murshudov, 2013) as implemented in the autoPROC processing package (Vonrhein et al., 2011). To determine an initial set of phases, a composite reflection file was generated by merging datasets derived from multiple crystals with KAMO (Yamashita et al., 2018). These multi-dataset reflections were then subjected to molecular replacement with the program Fragon (Jenkins, 2018) using three fragment search models comprised of ten-residue ideal α-helices. Following structure solution, ADP and coordinate model refinements were performed against a single crystal dataset using the program *phenix.refine* (Adams et al., 2010) in combination with manual real-space model building and refinement in the program *Coot* (Emsley & Cowtan, 2004). Collection and refinement statistics are available in Table S2. The structure has been deposited as PDB code 9C5S.

## Results and Discussion

To understand CV1 dimerization, we first sought to define the contribution of individual amino acid residues to dimerization. To do so, we prepared variants of CV1 in which each non-alanine, non-cysteine residue was individually substituted with alanine. In total, ten variants were prepared: T2A, F3A, N5A, L6A, W7Y, R8A, L9A, L10A, Q12A, and N13A. For Trp7, we opted to substitute with tyrosine instead of alanine to allow determination of all peptide concentrations by UV-Vis spectrophotometry. The R8A variant displayed limited aqueous solubility that prevented further study.

**Figure 1:**
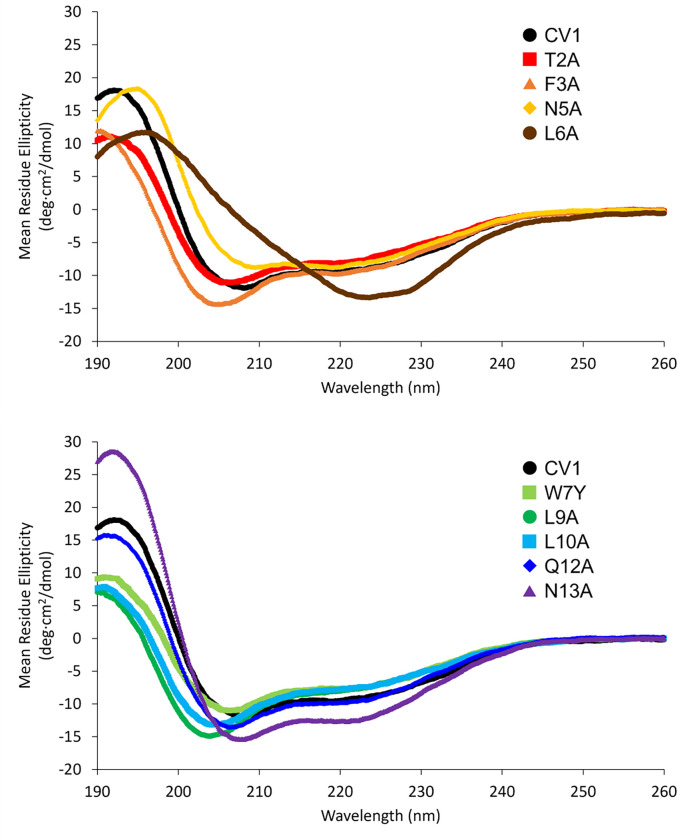
Circular dichroism spectra for the CV1 bisthiol peptide and variants. Spectra were acquired using a Jasco J-1500 CD spectrometer at a concentration of 30 μM in 10 mM sodium phosphate, pH 7.5 in a 0.1 cm pathlength cell at 25 °C

Analysis of bisthiol variants by CD spectroscopy revealed that all variants except L6A exhibited partial α-helical folding in sodium phosphate buffer. Aside from the L6A variant, all CD spectra were consistent with α-helical folding, displaying dual minima at approximately 208 and 222 nm (Fig. 1). Based on a calculated minimum mean residue ellipticity (MRE) of approximately − 27000 deg·cm^2^/dmol at 222 nm for an ideal 14-residue α-helix (Shepherd et al., 2005; Wang et al., 2006), the original CV1 bisthiol peptide is estimated to be approximately 30% α-helical in 10 mM sodium phosphate buffer at 25 °C, which is consistent with previous results (Victorio & Sawyer, 2023). Estimated α-helix content ranged from 25% for the L10A variant to approximately 45% for the N13A variant at 25°C. In contrast, the L6A variant has a maximum around 196–197 nm and a broad minimum between 220 and 230 nm, indicating a non-α-helical structure that is distinct from a random coil (Bishop et al., 2001).

Oxidation of bisthiol monomers under mild conditions produced dimers as the major product for many, but not all, variants. Dimerization was performed as previously described using a peptide concentration of 0.88 mM in 5 mM aqueous NH_4_HCO_3_ buffer containing 20% (v/v) DMSO as oxidant. Based on HPLC quantification, reactions with CV1 and three variants (N5A, Q12A, N13A) produced the dimer as the exclusive product. The W7Y variant also displayed significant preference for the dimer product (> 90%) over the competing intramolecular disulfide species. For four remaining variants – T2A, F3A, L9A, and L10A – a mixture of dimer and intramolecular disulfide products was observed. For the L6A variant, only the intramolecular disulfide product was observed upon oxidation, which is consistent with previous results suggesting the necessity of α-helical folding of the bisthiol monomer to yield dimers. When corrected for the estimated extinction coefficients of 11,000 M^− 1^·cm^− 1^ for the dimer and 5500 M^− 1^·cm^− 1^ for the intramolecular disulfide species, the ratios of dimer-to-intramolecular disulfide were 1:1 for the T2A variant, 2:1 for the F3A variant, 3:1 for the L9A variant, and 1:2 for the L10A variant. All dimers were determined to be antiparallel based on trypsin digestion (Figure S3). Based on these data, Leu6 and Leu10 appear to be major contributors to dimerization with additional contributions from Thr2, Phe3, and Leu9. Importantly, the minimal contribution of Trp7 contrasts sharply with the previously proposed model in which tryptophan residues interact at the dimer interface (Victorio & Sawyer, 2023).

**Figure 2:**
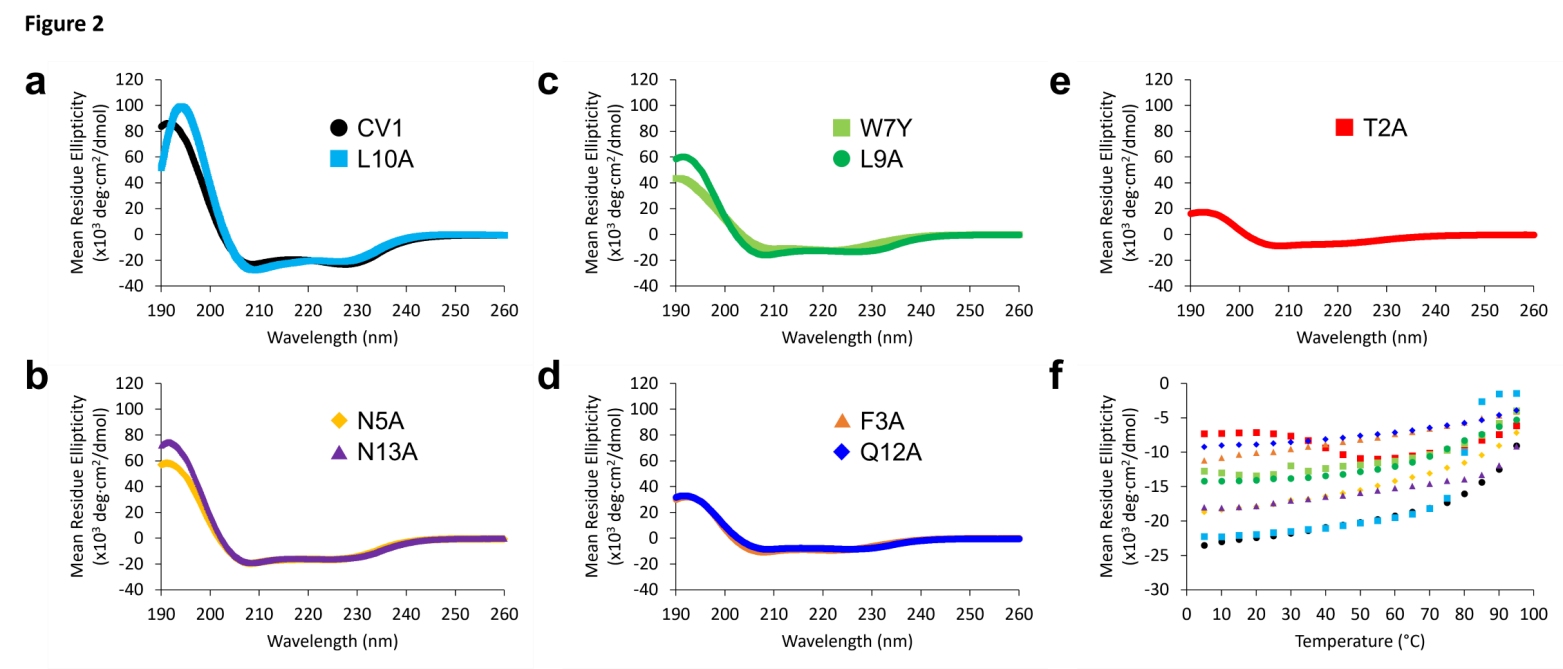
Circular dichroism spectra for the disulfide-linked CV1 peptide dimer and disulfide-linked dimers of CV1 alanine variants. Spectra were acquired using a Jasco J-1500 CD spectrometer at a concentration of 30 μM in 10 mM sodium phosphate, pH 7.5 in a 0.1 cm pathlength cell. a-e) Spectra at 25 °C. f) Mean residue ellipticity at 222 nm as a function of temperature (5 °C to 95 °C)

Evaluation of dimers by CD spectroscopy indicated that dimers formed from each variant exhibited different degrees of α-helical folding. At 25°C, the MRE at 222 nm ranged from approximately − 22,000 deg·cm^2^/dmol (70% α-helical) for CV1 to approximately − 7300 deg·cm^2^/dmol for the T2A variant (20% α-helical). The nine dimers (CV1 and eight variants) can be grouped into four pairs with one outlier (Fig. 2):

The first pair is CV1 and L10A, which were almost 70% α-helical at 25°C (Fig. 2A, black and light blue; Figure S4). Nonetheless, folding stability was starkly different for these peptides. The CV1 dimer shows gradual unfolding with an MRE of −9000 deg·cm^2^/dmol at 95°C, corresponding to approximately 50% α-helicity at this temperature. In contrast, the dimer of the L10A variant displayed a cooperative loss of α-helical folding between 75°C and 85°C (Fig. 2F, light blue). Thus, consistent with HPLC analysis, Leu10 appears to play a significant role in dimer stability.

Six of the remaining variants can be paired based on their α-helical folding as dimers at 25°C (Fig. 2B–D and Figure S4). At 25 °C, variants N5A and N13A were approximately 50% folded (−17,500 deg·cm^2^/dmol), variants W7Y and L9A variants were approximately 40% folded (−13,500 deg·cm^2^/dmol), and variants F3A and Q12A were approximately 30% folded (−9400 deg·cm^2^/dmol). The relative stability of these variants was maintained across the full temperature range up to 95°C, with retention of greater than 90% of their respective maximum folding up to 70°C.

Dimers of the final T2A variant displayed a highly unusual CD profile as a function of temperature (Fig. 2E–F, red; Figure S4). At 5–25°C, the MRE at 222 nm was relatively constant at approximately 20% α-helical (−7200 deg·cm^2^/dmol). As the temperature increases from 25°C to 55°C, the MRE at 222 nm *decreased* to approximately −11,000 deg·cm^2^/dmol, indicating an unusual increase in α-helical folding to approximately 40% in this temperature range. Beyond 55°C, the MRE for the T2A variant increased to −6200 deg·cm^2^/dmol at 95°C, approximately 40% of the maximum α-helical folding for this temperature range.

One important caveat to this analysis of relative α-helical folding of dimers is that all but three peptides (T2A, F3A, and W7Y) showed an apparent “shift” in the characteristic 222 nm CD minimum for α-helices to approximately 227 nm (Fig. 2). This shift was not observed for any of the bisthiol peptides, suggesting that the shift is related to dimerization. Aromatic-aromatic interactions, especially those involving tryptophan, frequently give rise to CD minima/maxima within this wavelength range (Andersen et al., 2006; Cochran et al., 2001; Nazzaro et al., 2023; Sawyer & Arora, 2018). This fact contributed to the previously proposed model involving tryptophan interactions at the dimerization interface (Victorio & Sawyer, 2023). The fact that the W7Y variant does not display this CD shift supports the hypothesis that the CD shift arises from interaction between tryptophan residues. The absence of this shift in the T2A and F3A variants was not initially clear from CD analyses but was subsequently rationalized based on the three-dimensional structure of the CV1 dimer (*vide infra*).

**Figure 3:**
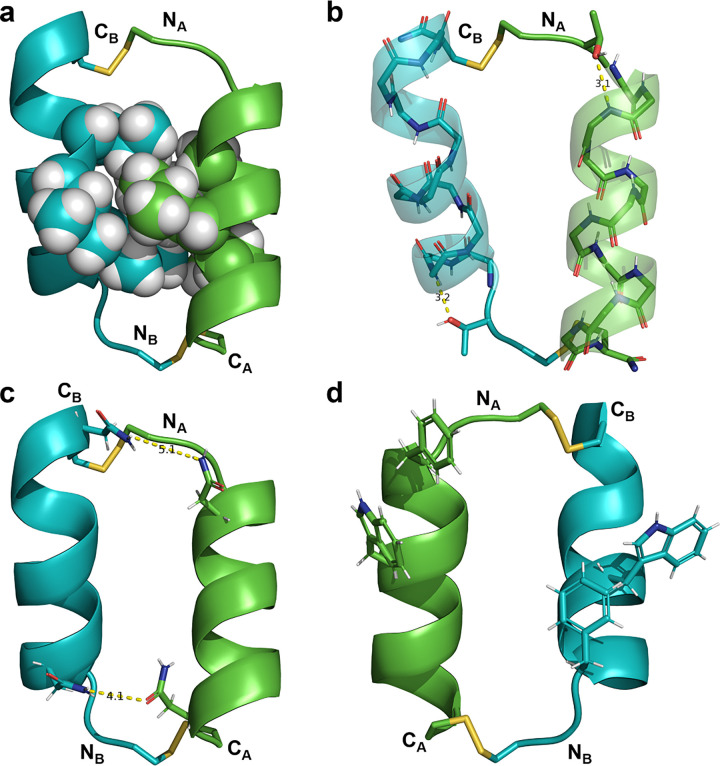
Crystal structure of disulfide-linked CV1 peptide dimer (PDB 9C5S). In all panels, each helix is represented with the same color (chain A = green, chain B = blue,) and disulfide bonds are represented as yellow sticks. N- and C-termini are labeled as N and C, respectively, with subscripts to indicate the corresponding chain. a) The dimer interface involves interdigitation of the side-chains of Leu6, Leu9, and Leu10 (spheres). b) The oxygen atom of each Thr2 side-chain serves as a hydrogen bond acceptor for Asn5 NH to cap the helix at the N-terminus. c) Asn5 and Asn13 side-chains form long range side-chain-to-side-chain hydrogen bonds between helices. d) Phe3 and Trp7 side-chains (sticks) are peripheral to the helical interface

Concurrent with alanine scanning and CD analyses, we also determined the three-dimensional structure of the CV1 dimer at 1.0 Å resolution. The asymmetric unit is composed of two interacting dimers (Fig. 3A). In contrast to the previously proposed dimer interface involving tryptophan residues (Victorio & Sawyer, 2023), the structure of each dimer in the crystal structure reveals a canonical knobs-into-holes packing between Leu6, Leu9, and Leu10 as expected for a dimer of α-helices (Hadley et al., 2008; Keating et al., 2001; Regan & DeGrado, 1988; Tripet et al., 2000). The helical portion of each peptide is capped at the N-terminus by Thr2 (Fig. 3B; (Aurora & Rose, 1998)). Cysteine residues extend beyond the helical segment of each peptide to form bridging disulfide bonds. Asn5 and Asn13 form long-range hydrogen bonding interactions within each dimer (Fig. 3C), which is consistent with the slightly decreased stability of the N5A and N13A dimers relative to CV1 in CD experiments. For each dimer, one of the Phe3 residues and both Trp7 residues are oriented away from the dimer interface, suggesting a limited role for these residues in dimerization/disulfide bond formation (Fig. 3D).

**Figure 4:**
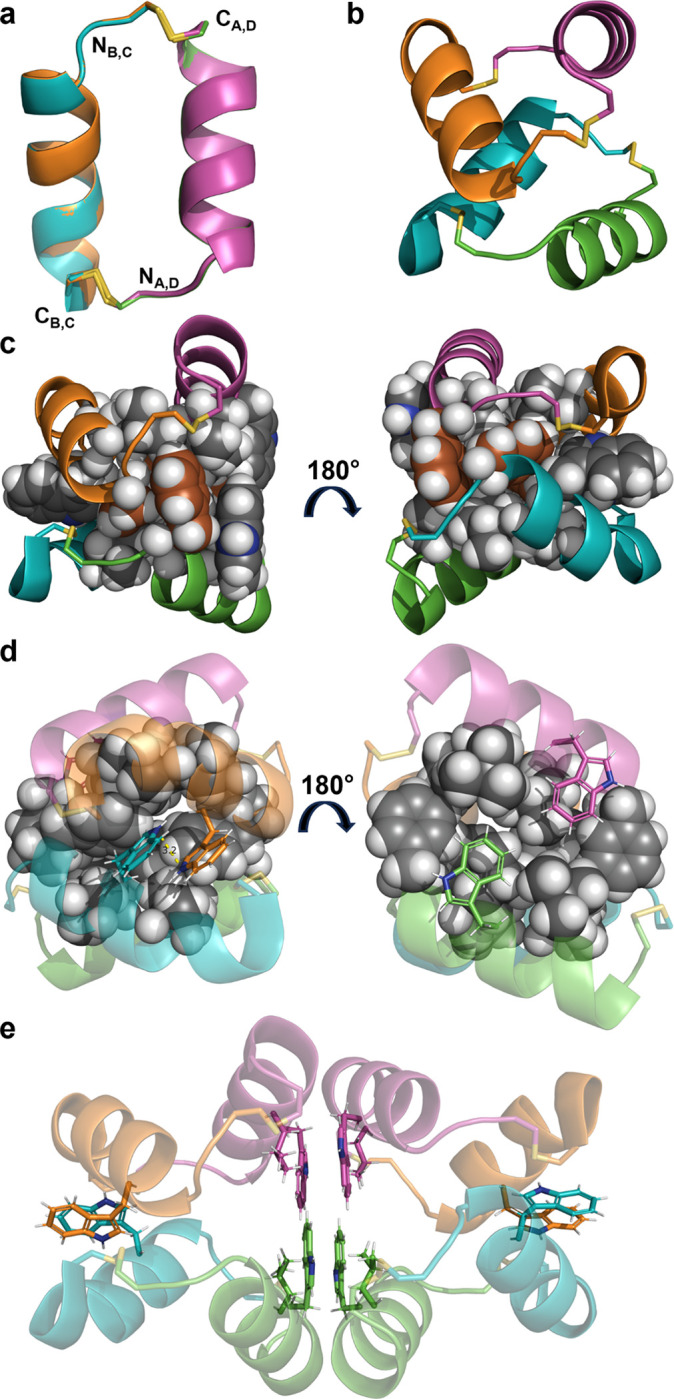
Analysis of crystallographic symmetry. In all panels, each helix is represented with the same color (chain A = green, chain B = blue, chain C = orange, chain D = pink) and disulfide bonds are represented as yellow sticks. N- and C-termini are labeled as N and C, respectively, with subscripts to indicate the corresponding chain. a) An overlay of the two copies of the dimer observed in the asymmetric unit shows high similarity (RMSD = 0.28 Å for all backbone atoms). b) The dimer of dimers is antiparallel with close contact between chains B (blue) and C (orange) and a wider interface between chains A (green) and D (pink). c) Phe3 residues (brown) cap an extended hydrophobic core formed primarily by Phe3, Leu6, Trp7, and Leu10 (spheres). d) Trp7 residues form two different arrangements. Between chains B (blue) and C (orange), Trp7 residues are separated by approximately 3.2 Å in a parallel displaced stacking arrangement. Between chains A (green) and D (pink), Trp7 residues are not interacting but pack in a parallel fashion against other hydrophobic core residues. e) The crystallographic octamer (dimer of dimer of dimers) is formed by extensive hydrophobic and π-π interactions between Trp7 and Leu10 from chains A (green) and D (pink) in adjacent dimer of dimers

While not contributing to individual dimers, the Phe3 and Trp7 residues appear to stabilize a dimer of dimers in the asymmetric unit and help to rationalize several observations from previous and current CD data (Victorio & Sawyer, 2023). The individual dimer copies within the dimer of dimers overlay well (Fig. 4A, RMSD = 0.28 Å for all backbone atoms). The arrangement of dimers is antiparallel and highly asymmetrical (Fig. 4B). The interface between dimers resembles a hydrophobic protein core and is composed of four copies each of Phe3, Leu6, Trp7, and Leu10. The Phe3 residues form interacting pairs on either end of the hydrophobic core with a specific packing arrangement in which Hβ atoms from Phe3 of chains B and C form apparent CH-π interactions (Nishio et al., 2014; Zondlo, 2013) with the ring faces of Phe3 from chains D and A, respectively (Fig. 4C). The Trp7 residues form two types of interactions: the Trp7 residues from chains B and C form a parallel displaced stacking arrangement at a distance of approximately 3.2 Å (Figure 4D, *left*) while the Trp7 residues from chains A and D are parallel to each other but interacting with other hydrophobic core residues instead of each other (Fig. 4D, *right*).

In particular, the parallel displaced stacking of Trp residues at the interface between dimers provides a rationale for the apparent shift of the 222 nm CD minimum to 227 nm, which is observed in the spectra of CV1 and many other variants (Grishina & Woody, 1994). As mentioned previously, aromatic-aromatic interactions, especially those involving tryptophan, frequently give rise to CD minima/maxima within the 225–235 nm wavelength range (Andersen et al., 2006; Cochran et al., 2001; Nazzaro et al., 2023; Sawyer & Arora, 2018). The absence of the CD shift for the W7Y dimer supports the idea that the CD shift is related to Trp-Trp interaction. The absence of the 227 nm CD shift for the F3A dimer can be rationalized by the apparent role of the Phe3 residues in stabilizing the dimer of dimers that results in Trp-Trp stacking. The reason for the absence of a CD shift for the T2A dimer is less clear but may be related to the capping function of Thr2, which presumably stabilizes individual helices to promote the formation of the dimer of dimers.

In addition to revealing the interactions driving dimerization of CV1 and its dimer of dimers assembly, the X-ray structure also revealed interactions that drive crystal packing. Residues Trp7 and Leu10 from chains A and D form a relatively flat face that forms hydrophobic and π-π interactions with the same residues from an adjacent dimer of dimers to form an octameric (or dimer of dimer of dimers) structure (Fig. 4E). This hierarchical assembly leads to a porous crystal structure with polar residues (Asn5, Arg8, Gln12, and Asn13) facing the pores, reminiscent of functionalized peptide-derived crystalline frameworks based on π-stacking strategies (Heinz-Kunert et al., 2022; Vijayakanth et al., 2024).

Overall, we report biophysical and crystallographic characterization of spontaneous peptide dimers based on the N-terminus of the p53 protein. CD spectral and structural data highlight major roles for the Leu6 and Leu10 residues in mediating interaction between peptide chains within each dimer. Phe3 and Trp7 residues promote higher-order assemblies of four and eight peptide chains, though such assemblies are not required for efficient dimerization. The four-chain dimer of dimers possesses a protein-like hydrophobic core, and its existence in dilute aqueous solution is supported by the 227 nm minimum observed for almost all Trp-containing variants.

Moreover, structural data suggests a designable molecular architecture beyond the p53 sequence. The all-leucine core at the dimer interface forms a LxxLL pattern, which is a consensus motif through which transcriptional coactivators bind to nuclear receptors in an α-helical conformation (Galande et al., 2005; Heery et al., 1997; Plevin et al., 2005). Beyond its role in transcription, the LxxLL motif is also found in the ubiquitin ligase UBE3A, also known as E6AP, which interacts with the E6 protein of human papillomavirus (HPV) to mediate p53 degradation as a hallmark of the HPV-related carcinogenicity (Ye et al., 2023; Zanier et al., 2013). Thus, it seems highly likely that other LxxLL motif-containing peptides could be dimerized in the same way as the CV1 peptide for potential protein targeting and/or intracellular delivery.

## Figures and Tables

**Table 1:** Peptide Naming and Sequences.

Peptide	Sequence
CV1	Ac-CTFANLWRLLAQNC-NH_2_
T2A	Ac-CAFANLWRLLAQNC-NH_2_
F3A	Ac-CTAANLWRLLAQNC-NH_2_
N5A	Ac-CTFAALWRLLAQNC-NH_2_
L6A	Ac-CTFANAWRLLAQNC-NH_2_
W7Y	Ac-CTFANLYRLLAQNC-NH_2_
R8A	Ac-CTFANLWALLAQNC-NH_2_
L9A	Ac-CTFANLWRALAQNC-NH_2_
L10A	Ac-CTFANLWRLAAQNC-NH_2_
Q12A	Ac-CTFANLWRLLAANC-NH_2_
N13A	Ac-CTFANLWRLLAQAC-NH_2_
